# Emotion Analysis Based on Deep Learning With Application to Research on Development of Western Culture

**DOI:** 10.3389/fpsyg.2022.911686

**Published:** 2022-09-13

**Authors:** Ming Chen

**Affiliations:** Centre for European Studies, College of Foreign Languages and Literature, Sichuan University, Chengdu, China

**Keywords:** deep learning, emotion analysis, BERT, BiLSTM, cultural development

## Abstract

Cultural development is often reflected in the emotional expression of various cultural carriers, such as literary works, movies, etc. Therefore, the cultural development can be analyzed through emotion analysis of the text, so as to sort out its context and obtain its development dynamics. This paper proposes a text emotion analysis method based on deep learning. The traditional neural network model mainly deals with the classification task of short texts in the form of word vectors, which causes the model to rely too much on the accuracy of word segmentation. In addition, the short texts have the characteristics of short corpus and divergent features. A text emotion classification model combing the Bidirectional Encoder Representations from Transformers (BERT) and Bi-directional Long Short-Term Memory (BiLSTM) is developed in this work. First, the BERT model is used to convert the trained text into a word-based vector representation. Then, the generated word vector is employed as the input of the BiLSTM to obtain the semantic representation of the context of the relevant word. By adding random dropout, the mechanism prevents the model from overfitting. Finally, the extracted feature vector is input to the fully connected layer, and the emotion category to which the text belongs is calculated through the Softmax function. Experiments show that in processing short texts, the proposed model based on BERT-BiLSTM is more accurate and reliable than the traditional neural network model using word vectors. The proposed method has a better analysis effect on the development of western culture.

## Introduction

Literary works are often important medium for carrying cultural developments. Through the analysis of literary works, we can effectively analyze the context of cultural development. At the same time, we can make in-depth judgments on the current cultural trend and future development direction. Therefore, through text emotion analysis of literary works, the cultural development can be analyzed and a relatively automated process can be realized. Sentence-level text emotion analysis, that is, sentence emotion analysis, is the process of analyzing, processing, summarizing, and reasoning on subjective texts with emotional colors. With the development of social media such as forums, blogs, and Twitter, we have a huge amount of emotion data. Emotion analysis technology plays an increasingly important role in modern society.

Existing emotion analysis techniques include emotion dictionary-based methods, machine learning-based methods, and deep learning-based methods. The methods based on emotion dictionary analyze the text by word and syntax, and calculate the emotion value as the basis for judging the emotion tendency of the text. These methods can well lock the text emotional information according to the emotional dictionary, and the implementation is relatively simple. When individuals express language, they will add necessary emotional vocabulary, emotional praise and derogation words, adverbs of degree, negative words, etc., which have an important role in promoting or weakening emotional semantics (Rosenmann, 2015). The work in Westjohn et al. (2017) expanded the emotion dictionary by constructing related dictionaries such as negative words, adverbs, emotional expressions, etc., which greatly enhanced the ability to judge the emotional polarity of texts. Authors in Chen et al. (2020) combined corpus and dictionary to construct an adaptive emotion dictionary to improve the polarity classification of emotion in Weibo. Settanni and Marengo (2016) used the initial emotion seed words to construct a domain emotion dictionary for Chinese social media texts, which effectively improved the emotion classification of sentence-level texts.

The methods based on machine learning mainly converts the emotion analysis of text into a classification problem, and then uses classic support vector machines (SVM), naive Bayes and other machine learning algorithms (Liu et al., 2016) to obtain a model through supervised training. Then, the trained models perform text emotion analysis. However, because the traditional machine learning algorithms mostly use the bag-of-words model to represent texts, they face the problems of sparse data features and cannot extract emotional information contained in texts well.

The deep learning methods that have emerged in recent years can better make up for the shortcomings of traditional machine learning algorithms. Deep learning models represented by convolutional neural networks (CNN) and recurrent neural networks (RNN) have been widely used in the field of text emotion analysis. Keshavarz and Abadeh (2017) used the CNN model to learn the deep semantic information in the text and mine the emotional tendency of the Twitter text, which has achieved a significant improvement over the traditional machine learning methods. Zhu et al. (2017) proposed a unified CNN-RNN model for visual emotion recognition. The architecture utilized multiple layers of CNN to extract features at different levels within a multi-task learning framework, and proposed a bidirectional RNN to integrate the learned features at different levels in the CNN model. This method greatly improved emotion classification performance as reported. Zhang et al. (2018) proposed a bi-directional Gated Recurrent Unit (BiGRU)-based hierarchical multi-input-output model, which considered both the semantic information and lexical information of emotion expressions. It achieved a breakthrough improvement in emotion classification of customer reviews. Researchers in Zhai and Zhang (2019) applied the fusion of BiGRU and attention mechanism to fine-grained text emotion analysis, and achieved good performance on different datasets. Since then, the hybrid neural network models based on CNN, BiGRU, attention mechanism, etc., have been widely used in text emotion analysis tasks (Cho et al., 2014; Mnih et al., 2014; Bahdanau et al., 2015; Vaswani et al., 2017; Minh et al., 2018; Zhou and Long, 2018; Adhikari et al., 2019; Xu et al., 2019). Word2vec (Mikolov, 2013) was currently the most commonly used word vector tool in the Natural Language Processing (NLP) field. The Glove model was proposed by Pennington et al. (2014), and it has become popular in recent years because it improved the training speed of word vectors on large corpora with high stability. A pretrained model is a deep learning architecture trained on a large benchmark dataset, on which subsequent tasks can be performed. Pretrained models are very helpful for improving many NLP tasks (Dai and Le, 2015). With the in-depth research of pre-training models, many NLP pre-training models such as ELMo (Peters et al., 2018), ULM-FiT (Howard and Ruder, 2018), and bidirectional encoder representations from transformers (BERT; Devlin et al., 2019) have been proposed one after another by pre-training a large text corpus as a language model. They created contextually relevant embeddings for each word in a given sentence, which will be fed into subsequent tasks. Choosing an efficient word vector representation tool has an extremely important impact on the application research of deep learning. Compared with traditional emotion classification methods, emotional words such as emotion words, negative words, and intensity words play a crucial role (Fu et al., 2013). Although emotional language words are very useful, the application of emotional language knowledge in deep neural network models such as CNN and long short-term memory (LSTM) in recent years were still very limited. Inspired by existing literature, this paper gives full play to the advantages of deep learning in text emotion analysis, and proposes a short text emotion classification model based on the fusion of BERT and BiLSTM. First, the BERT model is used to convert the trained text into a word-based vector representation. Then, the generated word vector is used as the input of the BiLSTM to obtain the semantic representation of the context of the relevant word. By adding random dropout, the mechanism prevents the model from overfitting. Finally, the extracted feature vector is input to the fully connected layer, and the emotion category to which the text belongs is calculated through the Softmax function. The experimental results show that the method in this paper has performance advantages compared with several existing text emotion analysis methods, and has strong application potential.

## Basic Theory

### Extraction of Emotional Information

The modifier dictionary generally includes negative words, degree adverbs, conjunctions, and other parts. When emotional words are surrounded by these modifiers, there is a high probability that the emotional polarity of the whole sentence changes, such as polarity reversal, strengthening or weakening, etc. Therefore, the comprehensive consideration of emotion words and modifiers is crucial for judging the emotion polarity of texts. In Lei et al. (2018), the authors used the method of constructing an emotional language database. The emotional language database constructed in this paper mainly considers emotional words, negative words and degree adverbs. Through the constructed emotional language library, the emotional information contained in the text sentence is extracted, so as to obtain the emotional information set corresponding to each text sentence. Some typical strategies are explained as follows:

Strategy 1: If the current word is an emotional word, the current word is directly added to the emotional information set.

Strategy 2: If the current word is an adverb of degree and the next word is an emotion word, the two are added to the emotion information set as a whole; if the emotion word already exists in the emotion information set, it is deleted.

Strategy 3: If the current word is a negative word and the next word is an emotional word, the two are added to the emotional information set as a whole; if the emotional word already exists in the emotional information set, it is deleted. Or if the negative word is followed by the adverb and the emotion word in turn, the three are added to the emotion information set as a whole. Similarly, if the whole of the adverb and the emotion word exists in the emotion information set, it will be deleted.

### Bidirectional Encoder Representations From Transformers

**FIGURE 1:**
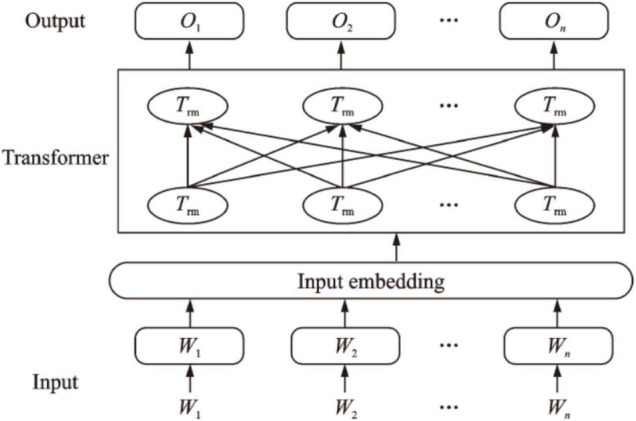
Basic struture of bidirectional encoder representations from transformers (BERT).

The BERT model is a pre-trained language understanding model based on multi-layer bidirectional Transformed model proposed by Devlin J (Settanni and Marengo, 2016) in the Google work team. The model is mainly composed of three parts: input layer, encoding layer and the output layer, whose structure is shown in Figure 1.

The BERT model innovatively proposes two representations at the word or sentence level. One is a character-level masked language model, and the other is a sentence-level next sentence prediction method. The introduction of the multi-head self-attention mechanism and the front-width neural network enables the BERT model to make full use of contextual features compared to other models, and convert the input corpus into a better feature representation.

### Bi-directional Long Short-Term Memory

**FIGURE 2:**
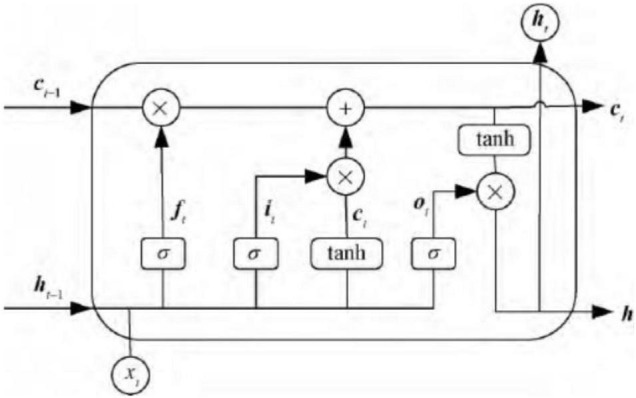
Basic struture of long short-term memory (LSTM).

Long short-term memory is a model of memory cell network structure proposed by Hochreiter to solve the phenomenon of gradient explosion and gradient disappearance in cyclic neural network processing relatively long time series data. It is based on RNN and introduces judgment information into cells. It meets the required threshold structure to control the accumulation speed of information from input gate, forget gate, output gate, so as to use this structure to memorize and update new information and solve the problem of long-term dependence. Each LSTM neuron is composed of a cell state, including a long-term state *c*_*t*_ and short-term state *h*_*t*_, input gate *i*, forgotten Gate *f*_*t*_, output gate *o*_*t*_. The basic structure of LSTM is shown as Figure 2.

The so-called cell state is a container for storing information. Through the process control of the input gate, the forget gate, and output gate, the information in the container is gradually increased, decreased, changed, and output. In each neural unit, the cell state undergoes the forgetting process of the forget gate, the input process of the input gate and the process of outputting information to the output gate. The input gate is to copy and process the input information of the current neural unit.

**FIGURE 3:**
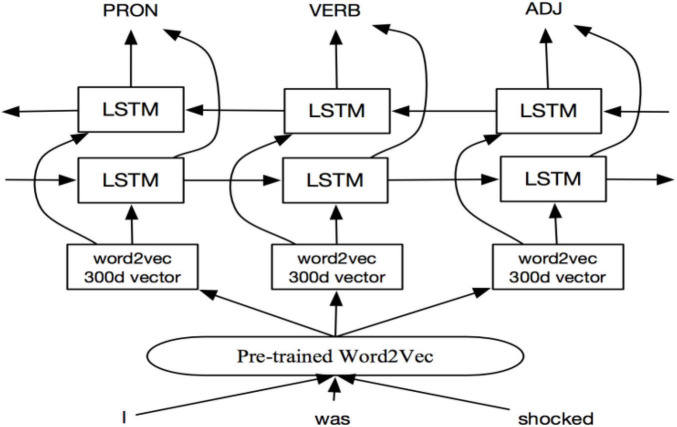
Basic struture of bi-directional long short-term memory (BiLSTM).

BiLSTM was a modified version of the traditional LSTM proposed by Liu et al. (2016). It appeared to solve the problem of LSTM in one-way processing. The model only analyses the “above” information of the text, and automatically ignores the “below” related information issues. The BiLSTM structure is composed of forward LSTM and backward LSTM, which has stronger memory ability than the traditional LSTM. Figure 3 shows the basic structure of the BiLSTM.

## Bidirectional Encoder Representations From Transformers-Bi-Directional Long Short-Term Memory Model

To properly handle the problem that the extracted feature vector space is sparse due to the short corpus of short texts, the short text emotion analysis model based on BERT-BiLSTM is proposed in this paper, which mainly composes of three parts, i.e., the text vectorized representation layer, BiLSTM layer, and emotion analysis discriminant layer.

### Text Vectorized Representation

The commonly used Word2vec (Keshavarz and Abadeh, 2017) is a text vector representation method with words as the processing unit. The process is cumbersome and requires text preprocessing, feature extraction, feature vector representation, and vector splicing. Finally, the vector representation of text can be generated. The accuracy of word segmentation in the processing stage directly affects the subsequent process and results. This paper focuses on the characteristics of short texts. The BERT model with the word as the processing unit is used to map each word in the text into a *k*-dimensional word vector form *x*_*i*_ ∈ R^*k*^, and input to other deep learning models. The form of text *X* after BERT vectorization is shown in Eq. (1):


(1) $$X=\left\{x_{1},x_{2},\ldots ,x_{i},\ldots ,x_{n}\right\}$$

where *n* represents the length of text *X*. *x*_*i*_ represents the *i*th word in vector form.

### Bi-directional Long Short-Term Memory Layer

The purpose of adding the BiLSTM layer is to allow the model to fully extract the emotional features of the text in combination with contextual semantics, which lays a good foundation for the next step to achieve emotional prediction. The vectorized text *X* = {*x*_1_,*x*_2_,…,*x*_*i*_,…,*x*_*n*_} is input to the forward propagation layer and backward propagation layer of BiLSTM, respectively. The forward propagation layer of BiLSTM will output the feature vector output by the forward hidden layer at each moment, and the final output vector set of the forward propagation layer is $\left\{{\vec{h}}_{1},{\vec{h}}_{2},\ldots ,{\vec{h}}_{n}\right\}\in R^{d}$, where *d* is the number of BiLSTM neurons, ${\vec{h}}_{n}$ represents the feature vector output by the forward hidden layer at the last moment. The backward propagation layer of BiLSTM will also output the feature vector output by the backward hidden layer at each moment. The set form is $\left\{{\overset{\leftarrow }{h}}_{1},{\overset{↼}{h}}_{2},\ldots ,{\overset{↼}{h}}_{n}\right\}\in R^{d}$, where *d* is the number of BiLSTM neurons, ${\overset{\leftarrow }{h}}_{n}$ represents the feature vector output from the backward hidden layer at the last moment. Afterward, the feature vectors of the last moments in both directions ${\vec{h}}_{n}$ and ${\overset{\leftarrow }{h}}_{n}$ are concatenated to form the feature vector of the final text *h* ∈ R^2*d*^, whose dimension is doubled. The final output of BiLSTM is shown as Eq. (2):


(2) $$h=\left\{{\vec{h}}_{n_{i}},{\overset{↼}{h}}_{n}\right\}$$

### Affective Tendency Discriminant Layer

The discriminant layer of the model is composed of the fully connected layer and the Softmax function. The feature vector *h* representing the entire text obtained by the BiLSTM hidden layer is input into the fully connected layer, and calculate the current text in each category. The probability is calculated as Eq. (3):


(3) $$\hat{y}=\mathrm{softmax}\,\left(W^{\text{T}}\,x+b\right)$$

where $\hat{y}$ represents the probability of the predicted category; *W* is a fully connected weight matrix of order *n* × *k*; *b* is the bias term, and *x* is the input feature vector.

### Training of Model

The loss function of the model is obtained by calculating the cross-entropy loss, and the model is trained by gradient descent. The calculation of cross entropy loss is shown in Eq. (4):


(4) $$L\,o\,s\,s=\frac{1}{N}\,\sum_{i=1}^{N}-\left[y_{i}\,\log\left(p_{i}\right)+\left(1-y_{i}\right)\,\log\left(1-p_{i}\right)\right]$$

In the above equation, *y*_*i*_ represents the label of the training sample *i*. The positive class of emotion in this paper is set to 1, and the negative class is set to 0. *p*_*i*_ denotes the probability that the model predicts the emotion tendency of training sample *i* as a positive class.

## Experiment and Analysis

### Datasets and Comparison Methods

For the research goal of western cultural development, this experiment selects the text dataset of literary works paragraphs, with a total of 40,000 pieces. Among them, the defined positive samples and negative samples account for 20,000, respectively. Before the experiment, the dataset is preprocessed, and then the dataset is divided into training set, validation set and test set according to the ratio of 8:1:1.

Since most of the short texts selected in the dataset are within 30 characters, the maximum length of the processed text of the model is set to 30 characters. The dimension of the BERT model is set to 768, and the number of neurons of the bidirectional LSTM is set to 128. The batch size is set to 128, and the dropout is set to 0.5 to prevent overfitting. In order to verify the effectiveness of the model, four other models are trained for comparison in this experiment, including CNN, LSTM, BiLSTM and BiLSTM + Attention. In particular, the BiLSTM + Attention method indicates that the attention mechanism is further introduced on the basis of BiLSTM. In order to effectively evaluate the performance of different methods, three evaluation factors are used including precision rate P (Precision), recall rate R and F-Measure.

### Results and Analysis

Various methods are tested on the selected dataset, and the statistics of the obtained results are shown in Table 1. It can be seen from the comparison that this paper obtains the best performance through the combination of BERT and BiLSTM. In particular, the performance improvement of the proposed method compared to the method directly adopting BiLSTM shows the important role of introducing the BERT model. Through the experimental comparison of BiLSTM and BiLSTM + Attention, it is found that adding the attention mechanism can further improve the analysis effect by enabling the model to obtain more internal related features of the text.

**TABLE 1:** Comparison of performance of different methods.

	Precision	Recall (%)	F-Measure
Proposed	0.912	0.914	0.905
CNN	0.852	0.843	0.829
LSTM	0.875	0.853	0.860
BiLSTM	0.893	0.862	0.874
BiLSTM + Attention	0.902	0.889	0.893

**FIGURE 4:**
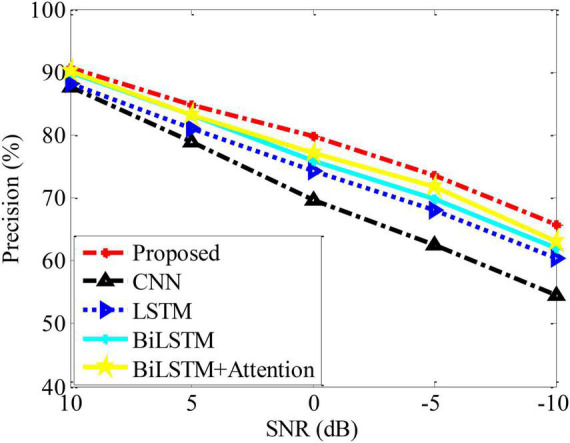
Performance of different methods under noises.

In the actual process, due to the influence of various external factors in the sample dataset, the prediction may cause certain errors in the analysis model. Therefore, the experimental data is subjected to a certain degree of noise condition to reflect the possible volatility of the samples. On this basis, using Precision as the basic evaluation index to test the performance trends of various methods, the results are shown in Figure 4. It can be seen from the figure that the performance of various methods is degraded to a certain extent due to the influence of noise. In contrast, the method in this paper can maintain the best analysis performance under different noise interference conditions, which further reflects its performance advantages.

## Conclusion

This paper takes text as the carrier and achieves the goal of western cultural development analysis through effective emotion analysis. A text emotion analysis model is developed based on the BERT-BiLSTM algorithm. Compared with traditional deep learning models, the proposed method is more efficient when dealing with short text tasks. Through the comparison of experimental results, it is found that the short text processing capability of the BERT-BiLSTM method proposed in this paper is better than some existing methods, and can achieve better emotion analysis goals. At the same time, it can be seen from the experimental results that the ability of text emotion analysis can be further improved by properly introducing the attention mechanism. Therefore, in future tasks, consider adding an attention mechanism to the model to further improve the performance of the model.

## Data Availability Statement

The raw data supporting the conclusions of this article will be made available by the authors, without undue reservation.

## Author Contributions

The author confirms being the sole contributor of this work and has approved it for publication.

## Conflict of Interest

The author declares that the research was conducted in the absence of any commercial or financial relationships that could be construed as a potential conflict of interest.

## Publisher’s Note

All claims expressed in this article are solely those of the authors and do not necessarily represent those of their affiliated organizations, or those of the publisher, the editors and the reviewers. Any product that may be evaluated in this article, or claim that may be made by its manufacturer, is not guaranteed or endorsed by the publisher.
